# Fracture Strength of Endodontically Treated Teeth Restored with Casting Post and Core and Glass-Fiber with Composite Core

**Published:** 2006-07-01

**Authors:** Sedigheh Saatian

**Affiliations:** 1 *Department of Prosthodontics, Dental School, Hamedan University of Medical Sciences, Hamedan, Iran*

**Keywords:** Casting Post and Core, Fiber Post, Fracture Strength

## Abstract

**INTRODUCTION:** Prefabricated metal and ceramic posts can be used with different kinds of core materials as an alternative to the conventional casting post and cores. It is unclear how these post and core systems can withstand different kind of forces in the mouth. The purpose of this study was to compare the fracture strength of endodontically treated, crowned maxillary incisors restored with casting post and cores and glass- fiber post with composite core and to evaluate their mode of fractures.

**MATERIALS AND METHODS:** Thirty caries free, human maxillary central incisors with incisoapical length of 23 ± 1 mm were divided into two groups. After root canal treatment procedures and decronation of teeth 2mm above cementoenamel junction, Grope 1 was restored with glass- fiber posts and composite cores and group 2 received casting post and cores. Teeth were prepared with a circumferential shoulder including a 1-2 mm ferrule and 0.5 mm bevel; all posts were cemented with an adhesive resin and teeth were restored with complete coverage crowns. Loads were applied at an angle of 135 degrees using a universal testing machine. Compression force was applied until the specimens fractured.

**RESULTS:** The median fracture strengths of groups 1 and 2 were 459 and 686 respectively (p<0.5). In group I, all fractures occurred in incisal third of roots. In groups II, 40% of fractures were in apical third and middle of roots.

**CONCLUSION:** Within the limitation of this study, the results suggested that glass fiber with composite cores can be used as an alternative to cast posts and cores in anterior teeth when creating 2mm ferrule effect was possible in normal occlusion. Clinical trial is required to verify these in vitro results.

## INTRODUCTION

In regards to the current trend of preservation of natural tooth structure with advancing age, the use of post and core restoration is increasing. In fact post and core restoration is used to restore functionality when the loss of tooth structure has been significant. The choice of an appropriate restoration for endodontically treated teeth is guided by tooth structure, root morphology, strength and esthetic. The cast gold post and core have been regarded as the “gold standard” in Post- and- core restorations due to its superior success rate (1,2). Alternatives to cast posts and core restoration have been developed. The use of prefabricated posts and custom made buildups with amalgam or composite simplifies the restorative procedures because all steps can be completed chair side (3).

All post and cores systems produce stresses within the root dentin, and cause fracture or bending of the post, root fracture, loss of retention or core fracture. Non tooth colored posts decrease the depth of the coronal translucency and the post may shine through in the cervical region, thus, altering the appearance of thin gingival tissue (4). Another problem with metal posts relates to the difference in modulus of elasticity between dentine (200 MPa) and these posts. This difference results in unequal distribution of strains on the dentin surface, and a tendency to create stress concentration areas (3,4). Nowadays different type of tooth colored post systems have been introduced to the restorative dentistry. They may be ceramic posts or Fiber reinforced post systems. These systems have improved the esthetics of teeth restored with full ceramic restoration. It is unclear how these new systems can withstand functional or Parafunctional forces in the mouth.

The present study evaluated the fracture strength of endodontically treated incisors restored with casting post and cores and glass fiber post with composite core.

## MATERIALS AND METHODS

Forty human maxillary central mc1sors with matured apices were obtained directly after extraction, restored in 0.1% thymol solution during the course of the study, which was less than 30 days. Teeth with cracks, caries, restorations and or roots shorter than 10 mm were discarded. Selected samples should have incisoapical length of 23 Q 1 mm. Endodontic treatment was performed through stepwise filing with reamers and headstrom files to International Standard Organization (ISO) size 60. Rinsing intermittently with 2.5% sodium hypochlorite solution, all roots were obturated with laterally condensed technique with gutta­ percha and zinc oxide eugenol sealer, the teeth were decoronated 2 mm coronal to the most incisal point of the proximal cementoenamel junction (CEJ) under continuous water cooling with a diamond bur, in a highspeed headpiece. All teeth received 1.5 mm shoulder finishing line preparations using a regular and fine tapered bur, in a highspeed headpiece, including a 1-2 mm ferrule. Post spaces were prepared by removing gutta-percha in such leaving 5 mm of root canal filling in the apical area. A N0.3 drill with diameter of 1.040 mm from the glass fiber post kit (Glassix, Swiss) was used to enlarge the root canals. Thirty teeth were selected based on the similarity of their root canal morphology and randomly divided into two groups.

Group I was restored with glass Fiber post kit (Glassix, Swiss) to create anchorage for composite core. In group 1, post space was cleaned and dried by ethyl alcohol, etched by 37% phosphoric Acid for 10 seconds , washed for 10 seconds with water , and then dried by air spray and paper point N0.40. Bonding liquid (single bond, 3M ESPE, USA) was applied to canal by microbrush. The excess of bonding liquid was removed by paper point and cured by light cure unit for 20 seconds. Posts were luted with chemically polymerized resin cement (Rely x Arc. 3M, ESPE, USA). Composite material (Filtek P60, 3M, ESPE, USA) was used as core material to yield an abutment to height of 4mm measured from the buccal shoulder. The convergence of approximately 6 degrees was prepared for abutments. 0.5 mm bevel were prepared around teeth to maximize ferrule effect.

In group 2, a custom cast dowel core was prepared by direct technique (5). Acrylic resin (Duralay, Reliance Dental, worth, Il) was used to fabricate the models. The core characters, such as length and tapering, were similar in the two groups. The casting posts and cores were also luted with a chemically polymerized resin cement (Rely x Arc. 3M, ESPE, USA) in the same group. Impressions were made for all teeth with the use of vinylpolysiloxane impression material (Extrude®, kerr, Italy), putty and wash system, and poured by type IV stone. Full veneer metal crowns were prepared and luted with a chemically polymerized resin cement (Rely x Arc. 3M, EPSE, USA).

To simulate a periodontal membrane, all roots were covered with a 0.1mm thick layer of auto polymerizing vinylpolysiloxane impression material (Extrude®, kerr, Italy), light body, and embedded up to 2mm beneath the CEJ in cold curing resin, In fact after the removal of any excess silicon, the silicone stent was used to position the teeth in a specimen holder, which was filled with auto polymerizing acrylic resin. The samples were loaded with a universal testing machine (lnstron 8503, Instron Group, Great Britain), at a crosshead speed of 1 mm/min until the fracture was taking place. Loads were applied at an angle of 135 degrees in the middle of lingual surfaces of the samples. Fracture loads and modes were recorded. Leveneʼs test was used to evaluate the equality of variance between two groups. As two sample groups have equal variance, therefore t­ Test was used to compare fracture loads among the two groups after the static load test.

**Table 1:** Comparison of the fracture strength of endodontically treated, crowned maxillary incisors restored with casting post and cores and glass-fiber post with composite core.

**Restoration type**	**No**	**Mean**	**Median**	**Min**	**Max**	**SD**	**Variance**	
Group I: (Glass-Fiber)	15	459.06	437	168	826	178.80		31972.4
Group II: (Metal)	15	686.133	638	257	1238	301.37		90824.2
Total	30	572.60	572.50	168	1238	269.47		72615.4

Tooth fractures were classified as repairable and catastrophic failures. (Fractures were classified as restorable if it was located in the incisal third of the root and catastrophic if located apical to that point) .As some of the samples were lost due to the mode of fracture, they did not allow removal of the teeth from acryl. Twenty teeth (10 from each group) were used to evaluate the mode of Fracture.

A Fisher exact test was performed to detect group differences in fracture mode. A significance level of P<0.05 was used for all comparisons.

## RESULTS

Table 1 shows the fracture strength of two groups. Group I exhibited lower strength values. Leveneʼs test showed equal variance for two sample groups. Therefore the t test was used which detected significant difference between two groups (P<0.05).

The fracture patterns of two groups are presented in Table 2. It showed that all catastrophic fractures belonged to group II, metal cast post and core. As there was no catastrophic fracture in samples of group I, the number of catastrophic failures was less than 5 in group II, and analyzed by Fisher exact test (P<0.05).

## DISCUSSION

In the case of substantial horizontal loss of the clinical crown, there was no restorative alternative to fabricating a post and core build up.The current study attempted to compare the conventional metal post and core with newer restorative approaches, glass fiber post. In this study all teeth have been selected based on their root length, and canal diameter, thus variations in posts length and diameter were not significant. All teeth received the benefit of 2 mm ferrule effect, which was clinically recommended for higher success rate .To produce a cushioning effect, all roots were covered with a 0.1mm thick layer of auto polymerized silicon, like the study of Martinez-Insua (6) and Akkayan (7). Guzy and Nichollas stated that force must be applied to anterior teeth in 130 degree angulations in vitro studies to reproduce the mouth situation in cl.I Angle occlusion (8).

**Table 2:** The fracture patterns of endodontically treated, crowned maxillary incisors restored with casting post- and– cores and glass- fiber post with composite core.

**Restoration ** **type**	**CF** ^a^	**FF** ^b^	**Total**
Group I: (Glass-Fiber)	0	10	10
Group II: (Metal)	4	6	10
Total	4	16	20

^a^ ***:*** *Catastrophic Fractures which located below of the incisal third of the root*

^b^ ***:*** *Favorable Fractures which located in the incisal third of the root*

The compressive force was applied at a crosshead speed of 1 mm/min until fracture.

Helkimo et al. reported that compressive force was 100-200 N. in anterior region of mouth (9). Ram fjord and Ash estimated mastication forces about 70-150 N in anterior teeth (10). Although in this study was the fracture strength of glass fiber posts was lower than casting posts, it was high enough to withstand mastication forces in anterior teeth. Plasmans et al. showed the same result in their study (11). But this study did not cover the effect of fatigue on this post system. In this study, the mode of fracture of two groups was different.

In group I, all fracture occurred in incisal third of roots. In fact they were repairable in clinical situation. In groups II, 40% of fractures were in apical and middle third of roots. Heydecke et al. reported that fractures would commonly occur in the apical half of the roots if rigid post systems were applied (3).

## CONCLUSION

This study showed that:

1. Glass fiber posts can be used in anterior teeth when creating 2 mm ferrule effect was possible in normal occlusion.

2. This restorative procedure can be used in patients without any parafunctional activity.

This study did not consider dynamic loading and the effect of thermocycling. In clinical situation, preparing casting post and core is more difficult for bicuspids with two divergent canals and in many clinical situations it is not possible to produce ferrule effect. Therefore it is recommended to perform more in vitro and in vivo studies to be designed.
